# Muslim patients in the U.S. confronting challenges regarding end-of-life and palliative care: the experiences and roles of hospital chaplains

**DOI:** 10.1186/s12904-023-01144-1

**Published:** 2023-03-27

**Authors:** Robert Klitzman, Gabrielle Di Sapia Natarelli, Elizaveta Garbuzova, Stephanie Sinnappan, Jay Al-Hashimi

**Affiliations:** 1 Vagelos College of Physicians and Surgeons, Joseph L. Mailman School of Public Health, Columbia University, 1051 Riverside Drive; Mail Unit #15, New York, NY 10032 USA; 2 Columbia University, New York, NY USA

**Keywords:** Religion, Coping, Spirituality, Chaplains, Islam, End-of-life care, Pain management, Hospice, Healthcare communication

## Abstract

**Introduction:**

Hospital chaplains aid patients confronting challenges related to palliative and end-of-life care, but relatively little is known about how chaplains view and respond to such needs among Muslim patients, and how well.

**Methods:**

Telephone qualitative interviews of ~ 1 h each were conducted with 23 chaplains and analyzed.

**Results:**

Both Muslim and non-Muslim chaplains raised issues concerning Islam among chaplains, doctors and patients, particularly challenges and misunderstandings between non-Muslim providers and Muslim patients, especially at the end-of-life, often due to a lack of knowledge of Islam, and misunderstanding and differences in perspectives. Due to broader societal Islamophobia, Muslim patients may fear or face discrimination, and thus not disclose their religion in the hospital. Confusion can arise among Muslim patients and families about what their faith permits regarding end-of-life care and pain management, and how to interpret and apply their religious beliefs in hospitals. Muslims hail from different countries, but providers may not fully grasp how these patients’ cultural practices may also vary. Chaplains can help address these challenges, playing key roles in mediating tensions and working to counteract Muslim patients’ fears, and express support. Yet many Muslim immigrants don’t know what “chaplaincy” is and/or prefer a chaplain of their own faith. Muslim chaplains can play vital roles, having expertise that can heighten trust, and educating non-Muslim colleagues, providing in-depth understanding of Islam (e.g., highlighting how Islam is related to Judaism and Christianity) and correcting misconceptions among colleagues. Hospitals without a Muslim chaplain can draw on local community imams.

**Conclusions:**

These data highlight how mutual sets of misunderstandings, especially concerning patients’ and families’ decisions about end-of-life care and pain management, can emerge among Muslim patients and non-Muslim staff that chaplains can help mediate. Non-Muslim chaplains and providers should seek to learn more about Islam. Muslim patients and families may also benefit from enhanced education and awareness of chaplains’ availability and scope, and of pain management and end-of-life options. These data thus have several critical implications for future practice, education, and research.

**Supplementary Information:**

The online version contains supplementary material available at 10.1186/s12904-023-01144-1.

## Introduction

The number of Muslim patients in the U.S. has been increasing in recent years [1], raising critical questions about these patients’ experiences and how well their needs are being met. Unfortunately, Islamophobia has also been rising in the U.S. and elsewhere and has been associated with poor mental health and lower health-care seeking among Muslims [2]. Muslims in the U.K., on self-report questionnaires, had lower scores on indices of mental health and well-being than did Christians [3]. Literature on Muslim patients in the world, particularly in Muslim-majority countries, has highlighted several challenges they may face, especially in non-Muslim majority countries – e.g., regarding maintenance of dignity during death and dying, provider education and support of their faith [4]. Muslims may see suffering as redemptive and death as God's will, and seek physical and spiritual dignity. The whole family may be the decision maker, and poor outcomes can cause fears of shame in the community.^4^ Yet Islamic culture is diverse and not monolithic, but rather includes various divisions and sects (e.g., Sunni and Shia) across many countries, shaped by various cultures [5] that can affect the particular challenges that families face. In the U.K., for instance, South Asian Muslim patients may also face potential language barriers [6]. A case study of a Somalian refugee patient in the Midwest U.S. highlighted language barriers as well [7].

Critical questions thus arise about how chaplains and other staff view and respond to the experiences and religious and spiritual needs of Muslim patients in U.S. hospitals. Chaplains and spiritual care can play critical roles in palliative, hospice and end-of-life care, but relatively little is known about how and how well U.S. chaplains meet the religious and spiritual needs of Muslim patients – e.g., whether particular challenges arise and if so, what. We were able to find only one published U.S. empirical study, published in 2015, based on interviews conducted by Abu-Ras and Laird [8] with 10 Muslim, 16 Christian and 7 Jewish chaplains in the New York City area.^8^ This study suggested that chaplaincy generally focuses on Christianity and Judaism, and pays little attention to Islamic perspectives. Overall, chaplains in this study perceived similarities, but also differences in the pastoral care needs of, and approaches toward, Muslim patients, and disagreed whether they should approach Muslim and non-Muslim patients the same (i.e., using a "one size fits all" approach) or whether specific needs exist and should be addressed. In recent years, chaplaincy has been changing, increasingly emphasizing non-denominational approaches [9]. Yet Abu-Ras and Laird see limitations in a “one size fits all” approach for meeting the religious and spiritual needs of Muslim patients.^8^ In this study, several non-Muslim chaplains offered prayer mats and Qur'ans, and perceived negative stereotypes of Muslims among staff, and needs to be aware of Muslim taboos regarding certain male–female interactions. Yet interviewees felt that Muslim patients may reject chaplaincy services, and local imams were commonly not trained in pastoral care and did not routinely visit the sick. Chaplains also felt that their roles included educating medical staff about these patients' needs and concerns.

A Michigan study of imams suggested that they were involved with congregants' healthcare, encouraging healthy behaviors in sermons, performing rituals around illness, helping congregants make medical decisions and educating staff about Islam [10]. Yet these roles did not appear to include regularly acting as chaplains per se within hospitals (i.e., visiting patients) to offer general or specific religious support.

Critical questions thus remain regarding how hospital chaplains, whether Muslim or non-Muslim, including those from outside New York City, view and approach these challenges, how they proceed, and how best to overcome these tensions – why and how these challenges “play out” in varied institutions, whether other aspects of these issues emerge, and if so, how, and when, and how these issues get addressed. To develop effective educational and other interventions to assist Muslim patients, it is vital to grasp how chaplains view these domains.

Recently, we conducted an interview study of chaplains, which included both Muslims and non-Muslims, concerning how they view and experience a wide range of challenges and opportunities in their work, related, for instance, to obtaining clinically-important information [11], using rituals [12], determining lengths of visits and relationships with patients [13] and facing rejections from patients [14]. Crucial issues regarding Islam spontaneously arose, and are thus presented here.

## Methods

The study adhered to COREQ guidelines [15]. Briefly, as discussed elsewhere [16–19], the Principal Investigator (PI), who has extensive experience conducting qualitative research, interviewed 23 chaplains by phone for approximately one hour each.

Qualitative approaches can gather the full ranges and kinds of views and behaviors, and guide subsequent quantitative studies. From a theoretical perspective, Geertz has urged the examination of individuals’ lives and social contexts by attempting to understand their own points of view, rather than by imposing theoretical structures, in order to obtain a "thick description" [20].

We recruited participants through the Association of Professional Chaplains and word of mouth. Interested individuals contacted the PI. Participants were interviewed until reaching "saturation" (i.e., "the point at which no new information of themes are observed in the data" [21].

Research reported in this paper was performed in accordance with the guidelines and regulations outlined in the Declaration of Helsinki. The Columbia University Department of Psychiatry Institutional Review Board approved the study (#7969).

All participants provided informed consent. Before the interview, the PI asked if the participant had had an opportunity to read the information sheet, and if so, had any questions, which the PI then answered. Participants who had read the information sheet, had no remaining questions and agreed to participate then provided informed consent that the PI then documented. The PI then conducted the interview.

### Data availability

The interviewees discussed identifying details in their open-ended responses, concerning themselves and patients and other providers, and the process of de-identification would be difficult and complicated, so that the data are not publicly available on any websites at the moment. The datasets used and analyzed during the current study are available from the corresponding author on reasonable request.

### Instruments

We drafted the semi-structured interview questionnaire, drawing on the past literature on chaplaincy and on informational, pilot interviews. Questions probed interviewees' experiences, views and decisions. Table 1 lists questions asked of participants (See Supplemental Material).

### Data analysis

For the methods, we adapted key elements of "grounded theory "[22]. We audio-recorded interviews and had them transcribed professionally. Initial analyses were conducted during the period that interviews were being held, in order to inform subsequent interviews. After all interviews were done, trained research assistants (RAs) and the PI analyzed the data fully in two phases. First, we each examined independently a subset of transcripts to determine participants’ views and factors that shape these, and recurrent themes, which were then given codes. The RAs and PI read each transcript, systematically coding sections of text, assigning "core" codes or categories, such as particular religions of patients and of chaplains. The PI and RAs reconciled these coding schemes, developed independently, into a single scheme.

Next, the PI and RAs independently content-analyzed the interviews, identifying major sub-codes, and variations in the core codes. From the sub-themes that each coder discerned, we developed a single set of "secondary" codes and further refined a set of major codes, and assessed social and contextual factors – e.g., specific challenges Muslim patients confronted regarding pain management, end-of-life and misunderstandings of Muslims.

The core codes and sub-codes were then employed in analyzing all of the interviews. Table 1 presents several sample codes. Interview excerpts below illustrate themes that arose.

**Table 1:** Characteristics of sample (*N* = 23)

Variable:	Number:	Percentages:
*Gender:*		
Male	13	56.5%
Female	10	43.5%
*Race & Ethnicity:*		
Caucasian	18	78.2.%
African American	3	13.0%
Latino	1	4.3%
Other	1	4.3%
*Age:*		
Range	42–75 years	
Mean	63 years	
*Geographic Region:*		
Northeast	12	52.2%
Midwest	4	17.4%
Southeast	3	13.0%
Southwest	3	13.0%
West	1	4.3%
*Religion:*		
Protestant	6	26.1%
Catholic	4	17.4%
Christian, not otherwise specified	6	26.1%
Jewish	3	13.0%
Muslim	3	13.0%
Buddhist	1	4.3%
*Highest Degree Held:*		
Master’s	10	43.5%
Doctorate	5	21.7%
Bachelor	1	4.3%
Associate	1	4.3%
Unknown	6	26.1%
*Years Practiced as Chaplain:*		
Range	3–30 years	
Mean	18.8 years	

## Results

As seen on Table 2, 10 participants were women and 13 were men; 78.2.% were Caucasian, 13.0% African American and 4.3% Latino; the mean age was 63 (range 42–72). They were from throughout the U.S., with diverse religions; 21.7% had doctorates and 43.5% had Masters degrees; and their mean length of practice was 18.8 years (range 3–30).

**Table 2:** Sample codes

**Background of Chaplain**
• Socioeconomic:
◦ Gender identity
◦ Identity:
▪ Cultural/ethnical identity
▪ Race
▪ Geographic location of childhood
▪ Religious background
**Chaplains’ Activities**
• Challenges arising concerning particular types of patients:
◦ Adjusting to different views/religions
◦ Different religions/spirituality:
▪ Religious patients:
• Christian:
◦ Lack of knowledge
◦ Lack of resources
• Judaism:
◦ Lack of knowledge
◦ Lack of resources
• Islam:
◦ Lack of knowledge
◦ Lack of resources
• Hindu:
◦ Lack of knowledge
◦ Lack of resources
• Other (specify):
◦ Lack of knowledge
◦ Lack of resources
**Implications**
• Understandings of spiritual/religious issues:
◦ Content:
▪ Western religions:
• Catholic
• Mainline Protestant
• Evangelical
• Judaism:
◦ Orthodox
▪ Non-Western religions:
• Islam
• Hinduism
• Other
• Practice

**Fig. 1:**
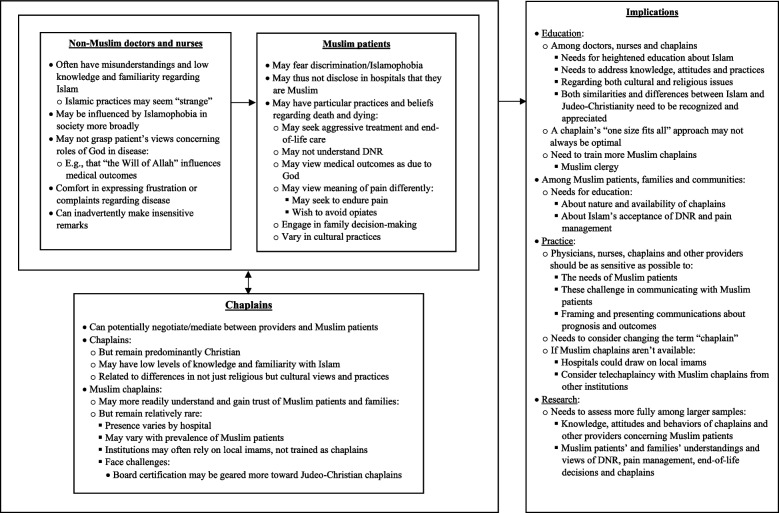
Themes concerning chaplains and Muslim patients

In brief, as outlined in Fig. 1 and described more fully below, in response to questions posed to all study participants concerning what challenges and impactful interactions they have encountered in their work, several participants spontaneously raised issues regarding Muslim patients and families. Additional probes asked about Islam as well. Both Muslim and non-Muslim chaplains discussed several topics concerning Islam that emerged among chaplains, doctors and patients, e.g., challenges and mutual misunderstandings between non-Muslim staff and Muslim patients, often due to limited knowledge and differences in perspectives. Several types, causes of, and approaches to, these challenges emerged. Muslim chaplains can bring particular background and understandings that can enhance trust and thus offer added benefits with certain Muslim patients’ needs.^8^

### Misunderstandings and discrimination

Stresses arise because Islam is widely misunderstood, and can be the subject of prejudice. As a Muslim chaplain said,

Islam is the most misunderstood religion in the world, especially in America. There is a lot of resistance from other faith traditions, which still have to understand where the Islamic community, persons, or faith come from in dealing with death and dying. Even chaplains from other faiths may be prejudiced against Islam. [Chaplain #3]

Certain particular aspects of Islam may fuel misunderstandings and feelings of lack of familiarity among non-Muslims. For instance, many non-Muslim Westerners may mistakenly feel that Muslim and Judeo-Christian notions of God are dramatically different, rather than related. As another Muslim chaplain said,

When we say 'Allah,' we're not talking about a Muslim God. We're talking about ***the Creator***. Muslims believe in God. It's that same God. It's just a different name. But people often think that we believe in some other kind of God. That's not true. We believe in the same One, as created Adam and Eve. So, when we're able to get them to understand that, it's a breakthrough. Non-Muslims may be put off in using the word ***Allah*** instead of ***God***. When they hear ***Allah*** it's strange to them. [Chaplain #21]

Yet non-Muslim staff frequently have little understanding of Islam, and are affected by social media, which can be biased. "They get their information from the news." [Chaplain #21].

Such limited knowledge, however, coupled with Islamophobia, can yield not only misunderstandings, but hesitancy and hostility. As a Muslim chaplain reported,

Sometimes we have a lot of resistance. One Christian student didn't know anything about Islam, and was saying all these negative things. I said, ‘I don't want to hear Muslims are this, Muslims are that.’ Even some very Orthodox rabbis I’ve met would just get up and walk out of the room if I talk about Islam and my theology. [Chaplain #3]

Relatedly, Muslim patients may also fear or face discrimination fueled by political events and certain politicians’ statements, and thus not disclose their religion to the hospital.

Since 9/11 and Trump, many Muslims also fear being misunderstood or facing discrimination. When asked their religion on hospital forms, some Muslims write ‘none.’ [Chaplain #21]

Muslim chaplains may therefore not rely wholly on hospital lists of self-identified Muslim patients, but proactively look for patients with Arabic names.

Islamophobia is not going down, especially in the last four or five years. [My city] is relatively better than most other places. But there is still fear. I see a lot of Muslim patients whose religion is listed as ‘unknown’ or ‘other’ because they don’t want to say they are Muslim. When patients are admitted, I look at their name and see if it’s Arabic. When I meet them, I find out. [Chaplain #22]

Muslim patients may thus be wary of discussing their religion unless chaplains are themselves Muslim or perhaps able to establish significant trust and sense of safety.

### Specific areas of misunderstandings

Misunderstandings can emerge regarding several specific issues, particularly regarding the role of God, pain, suffering, end-of-life care and family decision-making and interactions.

### The role of God in disease

Views can clash concerning the cause of individual disease and treatment outcomes, leading to problems in both *what* and *how* providers communicate. As a Muslim chaplain, reported,

Most non-Muslim doctors do not understand that Muslims believe that all events are due to the will of Allah. So, doctors saying to a patient's wife, 'Your husband has two months to live' rubs the family the wrong way, because they fervently believe that God, ***not*** the doctor, makes decisions about life. The physician should say instead: 'Among patients with your husband's condition, 80% live for around two months.' [Chaplain #21]

This view of God can have several implications, including affecting patients' acceptance of pain, and comfort expressing anger, frustration and negative feelings about their disease. Muslim patients may hence be relatively less open about their feelings – and just say they're fine, and a chaplain may therefore need to visit a few times to engage them. Such patients may feel that they cannot question their fate, which they see as derived from God.

They may think it’s not ok to complain because 'I have to be faithful to God. ***I have to be thankful no matter what situation we are in.’*** So, if that’s the issue, I share with them that people in the scripture Abraham, Moses, Jesus and Muhammad – all had life situations like this, very vulnerable moments, and expressed their vulnerabilities and shared their moments of sorrow and grief. So, it’s normal. Being angry with illness is ok. Moses was angry with his brother, Aaron. The Prophet Muhammad was sad, too, with the way he was being treated, but God counseled him. [Chaplain #22]

Chaplains may therefore draw on the Qur'an to show how historical religious leaders, too, were angry and sad, normalizing and validating these emotions for patients and families.

It is very difficult for Muslim patients to open up so quickly. It takes a while. They don’t want to share things. People from [certain Muslim countries] don’t like to share. To them, it’s private, special, they don’t want to make it public. So, it takes a while to make them open up. South Asian and East Asian people are also more likely to be very quiet and not want to share their emotions. So, as a chaplain I struggle with this. I also share with my colleagues that if you visit a Muslim patient, bear that in mind that even if they are dying they will say, ‘I’m ok, I’m fine.’ It takes a while; you may need several visits. [Chaplain #22]

### Pain management and end-of-life care

Problems surface because of differing views of suffering and its role. As a Muslim chaplain described,

The Prophet Mohammed teaches that ***it is virtuous to suffer***. Hence, this life is a trial-and-error for the next one. The Prophet also says that for every illness there is a cure, and that saving life is the most important thing. So, Do Not Resuscitate [DNR] orders and withholding of care pose problems. Many patients think that withholding ***some*** care means withholding ***all*** care. Doctors should frame it in other ways. For instance, Arab legal scholars say that no one can inflict harm or suffering on a patient. Risky treatments and procedures that have side effects and little, if any, chance of success can in fact make patients suffer. Patients and their families may accept ***that*** reason as a way to avoid additional futile interventions. [Chaplain #3]

Muslim patients themselves may thus misunderstand DNR, and benefit from education about the religion's stances on this issue.

Islam can also shape patients' and families' views and decisions about pain management.

Part of being a patient is being ***patient***. So, when a doctor asks, 'How are you doing?' Muslim patients may say, 'Ok,' even if they’re in pain, because it is virtuous to suffer – they will be rewarded because of dying, death, or suffering. At the end of life, it's important that many Muslims might not want a lot of morphine, if they can help it, because they want to be able to declare at death: 'there's no God, but the one God, and Mohammed is His Prophet.' Just as we would do certain rituals for Catholics, we want the patient to say that. [Chaplain #3]

Muslim patients with serious pain may, therefore, want to avoid opiates. As one Muslim patient told a chaplain,

I'm dying, and know I'm dying. I can't stand the pain, but don't like morphine because it makes me groggy, and when I die I want to be able to make a [Declaration of Faith]. [Chaplain #3]

Muslim chaplains can, however, aid such patients by reinterpreting the term, "declaration" more broadly and flexibly, pointing out that the Qur'an also supports patients not unduly suffering.

If patients are intubated and can't talk, we want them to raise their right index finger, or we will raise their right index finger, and then the family will say these words. If the imam is not there, it's the family's obligation to make that happen. It's important for the staff to know that. Chaplains and staff can tell the family, 'Look, the physician said you can go ahead and make this end-of-life ritual for this patient while he or she is dying. If the patient comes back to life, then we can do it again, and again.' [Chaplain #3]

Providers can thus learn to communicate about these topics and ways of interpreting and adopting practices in helpful manners. Staff can assist by re-framing DNR, hospice and palliative care as acceptable in Islam, by citing not only Arab legal scholars, but verses from the Qur’an itself.

Most Muslims do not understand DNR, so we try to explain what it means – to allow 'natural death.' Muslim patients misunderstand the language, and may think that DNR is against the religion, so I give verses from the Qur'an that say allowing natural death is ok. We don't look at illness and death as a ***bad*** thing, that you're being punished in most cases. We try to give Qur'anic verses, and sayings of the Prophet to patients that feel that, so they can make their own decisions. Muslim patients may require and benefit from particular approaches, based on recognizing their religiousness. [Chaplain #22]

Yet physicians may not grasp the implications of these beliefs. Another Muslim chaplain described a Muslim woman who, for instance, wanted more aggressive treatment for her dying husband, though the doctors considered such efforts futile. In the husband's chart, the staff wrote that she was ‘in denial,’ had poor coping skills, and wouldn't accept his situation. After several weeks of mutual frustration and antagonism, a social worker finally arranged for a Muslim chaplain to visit. The wife, it turned out, believed that without the treatment, her husband wouldn't go to heaven. The staff had failed to appreciate her perspective. This chaplain told her that God wouldn't want her dying husband to undergo more suffering, which additional treatment would cause, and she agreed.

### Family decision-making and interactions

Chaplains observed how they and other providers needed to be sensitive to not only religious, but cultural differences.

I tell doctors that culturally, decision-making might also be different. The family makes the decision, and hierarchy is involved. The grandmother or grandfather may be in Saudi Arabia, but helping to decide for the patient here. Physicians want to know who is making the decision, and rely on the patient to make it, but the patient has to get back to them. Doctors don't understand that the elders back in Pakistan are involved. I try to buffer this, and give doctors a handout to educate themselves. [Chaplain #3]

Doctors may also find it frustrating that family decision-making differs from Western medical ethics' focus on individual autonomy.

Here [in the West], we work differently. Western medical ethics emphasizes patient autonomy, so family decision-making is challenging for the medical team. They don't know how it works in another part of the world. [Chaplain #22]

### Diversity within Islam

Muslims hail from different countries, but providers may also not fully grasp how these patients’ relevant cultural practices may in fact range widely.

I’ve seen Muslim patients from all over the world, including the African-American community, and others who have gotten away from their cultural identity and want to be more Americanized. I try to focus on the individual, what their cultural needs are, whether they are Sunni, Shia or African-American. All of us are equal in prayers, religious services, washing before prayer and liking halal food, if available. But I try to navigate and work with patients and see what their cultural needs are. [Chaplain #3]

Islamic sects have similarities as well as differences. They all follow the Qur'an and the Prophet, but differences may arise due to culture or politics.

### The roles of Muslim vs. non-Muslim chaplains

Questions arose regarding the potential use of, and needs for, Muslim vs. non-Muslim chaplains with Muslim patients. Non-Muslim chaplains generally felt they were able to aid Muslim patients. As a Christian chaplain observed,

Some Christians say, 'Islam is based on the Ishmael and Isaac problem, so Muslims are bitter and always will be.' But every religion has fundamentalists. I have a hard time dealing with Fundamentalist Baptists. But overall, Muslims are very easy to work with. They're very peaceful, very intelligent and really good. When you embrace and reach out to them, they embrace you back. [Chaplain #8]

Chaplains commonly see varying religions as simply different roads to the same place. As a Protestant chaplain explained,

As Muslims, Jews and Christians, we are all praying to the same God. If I drive to my state capital, I can take the interstate, but other people may want to take the backroads. We're all going to get there, but in different ways. [Chaplain #2]

Non-Muslim chaplains may try to make even small comments or gestures to counteract Muslim patients' and families' fears, and express openness and support. As a Christian chaplain said.

With Muslims, I say '***as-salamu alaykum*** (peace be with you), and they respond, '***wa-alaikum-salaam*** (peace be upon you also).' All of a sudden, they're relaxed. They fear that we think they're all terrorists. They're not! [Chaplain #2]

Yet many Muslim immigrants don't know what "chaplaincy" is, and chaplains must thus first explain.

We meet a lot of immigrants and people who are not familiar with the term ***chaplains***. So, we have to give a little bit of introduction about what we do: that we address spiritual and emotional care. Then they understand. [Chaplain #22]

Muslim patients may hence have little familiarity with chaplaincy or see it as potentially aiding them. As a Christian chaplain said,

Muslims assume that the chaplaincy service isn't for them. They wouldn't necessarily know – America has a lot of anti-Muslim stuff going on right now. They'd be surprised to learn we have a Muslim chaplain, and Jum'ah prayer services and a box of prayer rugs and supplies. [Chaplain #9]

Mutual wariness and misunderstanding can consequently emerge between Muslim patients and non-Muslim providers.

Muslim patients may still, however, strongly prefer having a chaplain of their own faith.

A Muslim patient was dying at a major hospital, which sent him a Catholic priest. The Chaplaincy Department marketed themselves as a 'multi-faith' organization, but was not, because it left out Islam, Buddhism, Hinduism and others. The patient said, 'I do not want a Catholic chaplain. I want a Muslim one.' [Chaplain #3]

Muslim patients appear very grateful to have a chaplain of their own faith, whom they may trust more, feeling more understood. As a Muslim chaplain said,

A few days ago, a Middle Eastern patient shook my hand, and said, ’Speaking to you has been the most comforting thing that has happened to me here in the hospital, because I can connect with you! You are an Imam. You would understand me.’ [Chaplain #22]

With Muslim patients, addressing religious issues *early on* in care can also be especially helpful.

A big challenge for all of us – physicians, and the religious community – is to be able to help religious patients and family members who have never before been in the hospital, and come to the end, and we bombard them with medical terms and issues, without giving them an opportunity to understand it from a layman's terms. They are confused. Then, you've got friction between the medical community, the family, and the religious community. That's why it needs to be brought in in the front end – as opposed to the middle or the back end: what are their religious preferences, how important is it to them, would they or a family member like to talk to a religious person? [Chaplain #3]

More than non-Muslim patients, many Muslim patients may have particular religious rituals, such as those involving prayer and handwashing, which may be difficult to follow and/or adapt within the context of hospitals and new disease-related physical limitations. Such patients may benefit from chaplains who can specifically help them adapt these practices, but these providers therefore need to have a knowledge of Islam.

I get a print-out of every Muslim that comes to the hospital, and my responsibility is to go and touch base with them, introduce myself, and let them know the services available for them, because we truly want them to practice in their tradition. Those who pray five times a day, and now hear the words’ cancer’ and ‘death,’ think they’re coming here to die. We want them to practice their faith, but many of them can’t get up to wash and do the rituals they need to do to pray. I can be with them, and try to help them understand that this is ok, we can do that. Educating the staff about those situations is also very helpful. [Chaplain #3]

Muslim chaplains suggested that they might be able to provide certain added benefits to Muslim patients and families (who may more readily trust and relate to chaplains of their own faith), bring deeper understanding of specific religious and cultural issues (which may be more intertwined than for many Judeo-Christian patients living in far more secular societies), as well as address broader familial religious needs.

A family from the Middle East had traveled all over the world for a 14-year-old son with a tumor on his neck that was bigger than a softball. The doctor called, telling me to talk to this family, because there's nothing further he could do for the child. The child would die on the operating table, attempting to remove the tumor. He was the parents' only child, and in their culture, it was important to carry the family name on. The child was very educated and articulate. He did everything a wonderful young man likes to do. The father said, 'I've been all over the world and paid all this money. Now I've come to America. I'm going home now, broke. I have no money.' And he left the mother and the child in America all alone with no support system. It was terrible. He just walked away. So, I visited the child every day, just to talk with him. He liked soccer. We watched the games, and talked about girls – the good things in life. I wanted to keep it normal. And after these sessions, we would pray together. He was very religious. One day he said to me, 'Imam, help me do one thing: Help my mother regain her faith in God so I can meet her in Paradise.'

Every time I went into the room, she would walk out the room, and just stand by the door with her hands crossed, mad and angry. Angry with God. But she would not go far. I talked louder with him, so she could hear. The next day, she stayed in the room, but didn't pray at the end of our session together. She raised her hands just a little bit, but still didn't pray. The third time, she raised her hands and began to pray. I looked at him and he looked at me. He was so happy that his mother was praying again. Three or four days later, he died.

About a month later, his mother called me from the Middle East and said, 'Thank you.' I said, 'For what?' She said, 'You stood by me and my child when nobody else would. My husband left me. The doctors and the nurses left us. But you came and stayed. Now I go to the mosque and pray and do all the things I used to. Thank you so much for that.'

That's more important to me than anything you can pay me. I see I made a difference. To this day, I'm still honored to remember that child and his mother, what they gave. I tell all our staff and patients: 'You give me something.' I just want to be present, and support you the best that I can.' One day, we're going to find ourselves needing that same type of support. Hopefully, we can give everybody that – to be present for people at their bedside, no matter who they are, what their circumstances or their background are. Because at the end, we're all one human spirit, we are connected no matter what. Everybody is going to experience illness, and death. It's not the illness we are fighting, it's more how we deal with it, treat and help each other. [Chaplain #3]

Facing end-of-life issues may thus raise, too, patients’ and families’ broader ongoing spiritual and religious needs. Full knowledge of the specific religion and culture can clearly aid both a mother and her child. This chaplain also developed vital trust. It is unclear whether a non-Muslim chaplain could have assisted a family member regain her faith in her religion in this way. The fact that the chaplain was Muslim may have been keyt.

Given Islamophobia, chaplains may emphasize commonalties across faiths. Yet trained Muslim chaplains possess important specific expertise, though remaining relatively rare. Hospitals often lack trained chaplains from outside Judeo-Christian traditions. Yet, within chaplaincy, Muslim chaplains face challenges, since the field has been largely built and structured around the needs and views of Judeo-Christian patients.

Board certification itself can be hard since it was established with Christianity and Judaism as models. It wasn't anything I was expecting, due to resistance from other faith traditions. It caused me a lot of stress in the beginning: how are we going to get board certified, because the processes were mainly for Jewish and Christian theological educations. I had to bring the Islamic theological education into that process, while receiving the clinical education from Jewish and Christian educators and physicians. [Chaplain #3]

Though having an in-house Muslim chaplain appears to have certain advantages, the presence of such chaplains appears to vary with the hospitals' size, patient population and geographic location. Several hospitals have a Muslim chaplain because they treat a relatively larger number of Muslim patients, but not all such hospitals have one. "We had a Muslim imam who was one of our candidates," one Catholic chaplain recalled. “He is now gone. But it was nice because we have a lot of patients from the Middle East. They connect themselves with the community resources here." [Chaplain #19].

As a Christian pediatric chaplain in a relatively large city admitted,

I wish I could recruit non-Judeo-Christian chaplains. Unfortunately, in this region of the country, I can't. But we do have a couple of community members – imams, rabbis and several Buddhist temples. [Chaplain #17]

Muslim chaplains note that they are absent from many hospitals, but also recognize that these departments have limited resources.

A lot of hospitals don't have Muslim chaplains. Chaplaincy is a very small department compared to any other department in the hospital. When I was interviewed for this job, the program director said, 'Ok. Be mindful that this is a secular hospital. So…' I told him, 'Yes.' Secular hospitals don't have too much money for this. [Chaplain #22]

### Needs for education of non-Muslim chaplains and staff

Broader needs for education thus remain. As a Christian chaplain observed,

There's more of a need, especially in cities, for training to include religions other than Christianity and Judaism that aren't included anywhere in the formal curriculum. We have a Diversity Week in the fall and the spring, celebrating different cultures. But that's all. [Chaplain #1]

Muslim chaplains can also play vital roles educating non-Muslim colleagues, providing in-depth insider understanding of this faith. As a Muslim chaplain said,

There's nothing like having someone who's actually a ***practitioner*** of the faith and can give insight into what it is that we do and believe, and recognize that ***we want the same things that other people want***: I want my children to be safe. We don't want to have to worry about our houses being robbed. [Chaplain #21]

Judeo-Christian chaplains may feel more comfortable working with Protestant, Catholic or Jewish patients than with Muslims. Muslim chaplains can often assist, by correcting misconceptions among colleagues, and highlighting, for instance, how this faith grows out of Judaism and Christianity, and is thus connected to these other traditions.

I try to work through that process without getting angry or upset, because most people don't understand that Islam is just an extension of the teachings of religions from Judaism to Christianity. But that took me aback. Because of my tenacity, I was able to be able to stay and stick with the hardship that goes on with that. [Chaplain #3]

At hospitals without a Muslim chaplain, non-Islam chaplains or providers can incorporate a local community imam. As a Muslim chaplain advised,

Chaplains should make sure the patient's family and their imam are involved or contact a Muslim chaplain. Interfaith chaplains should have a list of local mosque leaders. Family members have a particular mosque, and its imam or his representative can come and be with the patient, and help them guide through end-of-life issues. [Chaplain #3]

## Discussion

These data highlight how, in treating Muslim patients, non-Muslim staff can face challenges with which chaplains can help. Physicians, nurses and even many chaplains may have low levels of understanding of Islam, which can create mutual sets of misunderstandings concerning patients' and families' end-of-life care and pain management decisions and other areas. While prior literature has outlined certain key elements in Islamic medical ethics [23] (e.g., that DNR and withdrawal of care are acceptable and that patients should recite the testimony of faith before death), the present data suggest that confusion often persists among Muslim patients and families about what exactly their faith permits, and how to interpret and apply their religious beliefs in hospitals, especially regarding death and dying.

While Abu-Ras and Laird^8^ elucidated several challenges faced by Muslim patients and chaplains, the present data reveal several additional critical areas – e.g., how *dynamic processes* may be involved and tensions may arise from certain underlying causes, how different types of obstacles and misunderstandings emerge, how lack of familiarity and knowledge create *mutual* barriers, and how these issues can be better and more fully recognized and carefully and sensitively addressed.

These data highlight, too, how Muslim patients in U.S. hospitals can vary significantly. While prior research on Muslim patients appears to be largely from Muslim-majority countries^4^, 20% of Muslim patients in the U.S. are African-Americans, of whom 69% were born in the U.S [24]. Among African-Americans, 2% identify with the Nation of Islam**.**^24^ Muslim patients who were born and raised in the Middle East and/or have recently immigrated and may have spent limited time in the U.S. face particular barriers, regarding not only religion per se, but also culture, which can mutually affect staff interactions and perceptions. Future studies and discussions of Muslim patients in the U.S. should thus distinguish and examine differences between these groups.

These data suggest, too, how Muslim chaplains may possess particular knowledge about Islam that can uniquely aid patients and families. Muslim families may want more aggressive treatment for dying patients because they feel strong religious duties to do whatever they can to assist their family members. Yet a Muslim chaplain can instead suggest that God would also not want the patient to undergo more suffering, which additional treatment would cause. Providing education on details about Islam may, however, be stronger coming from a Muslim rather than a non-Muslim chaplain. Muslim chaplains may be seen as having more legitimacy or authority and thus readily obtain greater trust from frightened and/or wary families and patients.

Though in prior research, chaplains disagreed whether either a "one-size-fits-all" or a Muslim-specific approach was needed^6^, the present data underscore needs for *both* of these two perspectives, and ways in which both similarities as well as differences can exist between Muslims and other patients that staff should thus be aware of, and able to address. Prior research also stated that "several non-Muslim chaplains said that Muslim patients tended to refuse, reject or mistrust services that they offer," and that "non-Muslim chaplains might interpret such refusal to mean that Muslims to do not need or want chaplaincy services at all."^6^ But the current data suggest, instead, that non-Muslim chaplains seem very much aware of the needs for such services, and may see such refusals as due to patients' misperceptions of chaplains, rather than as indicating lack of potential needs for such services**.**

These data have several critical implications for future education, practice, and research for doctors, nurses and other providers, illuminating the challenges that they may face with Muslim patients and ways of addressing these. Non-Muslim staff and chaplains should seek to learn more about Islam and establish ways to connect with Muslim patients. Yet, medical schools generally appear to teach little, if anything, about Islam. One published educational intervention was found to improve understandings of Islam among 11 palliative care clinicians [25], though how long the intervention lasted, or whether it was attempted any other time is unclear. We have found no reports on whether any medical schools specifically mention or cover Islam in their curriculum, and if so how, to what degree and with what outcomes. The present data suggest that staff may benefit from having some sense about Islam, even if small, to help reduce ignorance, unfamiliarity, wariness or discomfort. As part of these efforts, cultural humility and patient-centered approaches are critical as well, avoiding bias and stereotyping in caring for patients from other cultures or religions with which one is relatively unfamiliar. Cross-cultural understanding can foster cultural humility and curiosity in medical, psychological, spiritual and religious care [26]. In these efforts, having Muslim patients, chaplains or providers present their own views and perspectives to trainees can be vital.

Muslim chaplains and clergy can therefore serve vital roles in educating non-Muslim chaplains and staff about this faith. For some foreign-born and/or immigrant Muslim patients, both religion and cultural practices can differ from prevailing Western ones and be deeply intertwined. Though chaplaincy seeks to be non-denominational, obstacles may arise in non-Muslim chaplains seeking to aid Muslim patients, partly due to accompanying *cultural*, not only religious differences. While chaplaincy may focus just on addressing spiritual and religious issues, particular cultural variations may arise, too.

A sample of primarily Christian patients felt no difference in having a chaplain of their same or a different specific faith [27]. But this study’s authors concluded that whether this finding extends to Islam as well remains unknown. Realistically, hospitals' willingness to have a Muslim chaplain may vary with the numbers of Muslim patients, which ranges. Still, some hospitals in urban areas with considerable Muslim populations lack a Muslim chaplain, and appear to feel that use of outside clergy are sufficient.

Muslim chaplains also suggested that they might be especially able to gain trust from certain Muslim patients. A chaplain of one religion can use non-denominational approaches with patients of other religions, but may have difficulty and succeed less in educating a patient about specific tenets of a religion other than the chaplain’s own. Jewish patients may, for instance, be less convinced or compelled by a Catholic chaplain informing them about the specifics of Judaism and vice versa.

These data therefore raise questions, too, about potential limits or obstacles in non-denominational approaches, which future scholarship can probe more fully. Chaplains should continue non-denominational, multi-faith approaches, but also recognize when a patient confronts issues specific to a non-Western faith about which they have limited familiarity, in which case a chaplain of that faith may be helpful. Non-Muslim chaplains should seek to be as aware as possible of such potential gaps and needs for improvement. In hospitals without a Muslim chaplain, patients may benefit from chaplaincy departments more fully including community-based Muslim clergy, who may be able to build critical trust with Muslim patients, especially concerning areas of heightened sensitivity. Non-Muslim chaplains did not appear to feel that they were significantly missing Muslim patients' needs. Yet Muslim chaplains may be able to help in ways that non-Muslim chaplains may not always fully appreciate.

Still, in the U.S. as well as other Western countries, widely perceived tensions often exist between Islam on the one hand and Judeo-Christian traditions on the other. Chaplains can play significant roles in bridging these chasms, but doing so may not always be easy, given prejudices and relatively low levels of knowledge about Islam that may persist, even among chaplains. Such education should address, in particular, several types of challenges that the present data suggest – e.g., concerning beliefs about the perceived role of God, religious rituals, family-decision making and interactions, pain management and end-of-life care.

These findings suggest needs for research, and a research agenda to examine, with larger samples, the views of both Muslim and non-Muslim patients, physicians, nurses, and chaplains: to investigate more fully how often and to what degree the challenges found here arise, and how they can best be addressed**.** Such studies should explore how much non-Muslim staff and chaplains know about Islam, whether non-Muslim chaplains can sufficiently gain Muslim patients' trust in order to educate them about Islam (e.g., its acceptance of DNR and pain management), and if not, how best to proceed, how much and what doctors need to know about Islam to interact optimally with Muslim patients, how such training can best be provided, and whether and how often Muslim patients prefer, or have better health treatment or satisfaction outcomes with Muslim, rather than non-Muslim chaplains. Studies can also assess experiences and outcomes using telechaplaincy with a Muslim chaplain from another institution, if a particular hospital does not have one and local Muslim clergy are unavailable. Importantly, patients and caregivers should be included in such future studies as well.

These data have several potential limitations. These findings are based on a sample of 23 chaplains, which is sufficient for qualitative analyses. However, further studies with larger samples can help elucidate these issues and factors that may be involved. Since this study was performed in the U.S., it may not be generalizable to all other countries (e.g., Muslim-dominant ones), but may be generalizable within the U.S. and possibly to other Western countries.

In sum, these data examine several critical aspects of chaplains’ views and interactions with Muslim patients, and have key implications for future practice, education and research. Non-Muslim chaplains and providers should seek to learn more about Islam, and establish ways to connect with Muslim patients. Muslim patients and families may also benefit from enhanced education and awareness of chaplain availability, benefits, and roles.

## Supplementary Information

**Additional file 1: Supplemental File Table 1. **Chaplains and Muslim patients

## Data Availability

The interviewees discussed identifying details in their open-ended responses, concerning themselves and patients and other providers, and the process of de-identification would be difficult and complicated, so that the data are not publicly available on any websites at the moment. The datasets used and/or analyzed during the current study are available from the corresponding author on reasonable request.

## Abbreviations

DNR: Do Not Resuscitate
PI: Principal Investigator
RAs: Research Assistants
