# Supplementary material for: Muslim patients in the U.S. confronting challenges regarding end-of-life and palliative care: the experiences and roles of hospital chaplains
Source: BMC Palliat Care. 2023 Mar 27;22:28. doi: 10.1186/s12904-023-01144-1 (PMC10041735; doi:10.1186/s12904-023-01144-1)
Supplement: Supplementary file 1 — Additional file 1: Supplemental File Table 1. Chaplains and Muslim patients [file 12904_2023_1144_MOESM1_ESM.docx]

**Table 1: Semi-Structured Study: Questionnaire**

Thank you again for participating in this interview portion of our study. As we discussed in the consent form, we will be asking about your views, feelings and experiences concerning chaplaincy and related areas.

**What is your current role?**

- Doctor: ___
- Nurse: ___
- Chaplain: ___
- Other: ___

**Background:**

Gender: M__ F__ Other__

Age**:____**

- Were you raised in a particular faith tradition? If so, which?

**Views of religion and spirituality among doctors and patients:**

- Have you seen religious and spiritual issues affect medical decisions? If so, where and how?
- Religion and spirituality among doctors:
  - Many providers seem wary of religious and spiritual issues. What do you think?
  - Have you witnessed any situations in which providers could have responded better to these issues and questions?
  - Do you see room for improvement? If so, where?
- Religion and spirituality among patients:
  - Have you faced any challenges ever regarding different beliefs among patients? If so, what?
  - Have you faced any challenges ever regarding patients with a non-Western belief system? What did you do? Could you have done anything better? If so, what?
  - How should hospitals and institutions be better prepared to assist these patients?
  - Have you ever faced any challenges with patients who say they are “None of the Above” when asked to indicate with which of the major religions they are affiliated (and hence called “Nones”) or say they are “Spiritual but not Religious” (SBNR)?
  - What are the biggest misunderstandings about Nones and SBNR?
  - Many Nones and SBNRs say that spirituality is an individual activity and that they do not need a community. Have you heard that? Do you agree?
  - Do you think chaplains could help more patients? If so, how?

**Views of existential issues:**

- Have you ever faced existential issues among doctors and patients (e.g., issues concerning the meaning of existence)? If so, how?
- What do you think is the relationship between psychological and existential issues?

**Views on end-of-life:**

- Have you faced challenges in addressing religious, spiritual or existential issues at the end-of-life with patients or families? Do you think other providers’ experiences have been similar or different?
- Have you witnessed Nones reassess their beliefs at the end-of-life?
- Have patients ever asked you to pray that they will die? How did you respond?

**Perceptions of needs:**

- Do you see room for improvement in how providers address religious or spiritual issues? If so, how?
- Have you tried addressing these issues? If so, how? Has it worked?
- What has helped you that might assist others facing similar challenges?
- If there were more chaplains, what do you think would happen? How might it help more patients?

**Views on challenges faced:**

- What are the biggest challenges you have faced regarding religious, spiritual and existential issues with:
  - Doctors: ___
  - Nurses: ___
  - Chaplains: ____
  - Patients and their families: ___
  - Other health care providers: ___
  - Hospital administrators/staff: ___
  - Institutions: ___
- Do specific cases/incidents come to mind? What have been the hardest experiences/cases you have had as a chaplain?
- Do you find resistance to these issues among:
  - Doctors: ___
  - Nurses: ___
  - Patients and their families: ___
  - Other health care providers: ___
  - Institutions: ___
  - Hospital administrators/staff: ___
  - Others: ___
- If so, when and ho much?
- Have you ever faced or addressed burnout among providers? If so, when? What happened?
- What other barriers arise in your work?

**For chaplains only:**

- How did you come to be a chaplain?
- How long have you been a chaplain?
- What kind of work did you do before?
- What kind of work do you now do as a chaplain?
- What have been your most rewarding experiences/cases as a chaplain? What were the most difficult? Do specific cases/incidents come to mind?
- How do you think chaplains can most help patients and their families? Physicians? Other staff? The hospital? The health care system?
- About what proportion of the patients you see are referred by:
  - Doctors: ___
  - Nurses: ___
  - Other: ___
  - None of the Above: ___

**Differences among chaplains:**

- Do you think other chaplains’ experiences have been similar or different? If so, how?
- Do you see differences between chaplains in different institutions or geographical regions? If so, how?

Thank you again for your participation.
